# Crowd-figure-pictograms improve women’s knowledge about mammography screening: results from a randomised controlled trial

**DOI:** 10.1186/s13104-018-3437-z

**Published:** 2018-05-21

**Authors:** Maren Reder, Lau Caspar Thygesen

**Affiliations:** 1 School of Public Health, Bielefeld University, Universitätsstraße 25, 33615 Bielefeld, Germany; 2 Institute of Psychology, University of Hildesheim, Universitätsplatz 1, 31141 Hildesheim, Germany; 3 National Institute of Public Health, University of Southern Denmark, Studiestræde 6, 1455 Copenhagen K, Denmark

**Keywords:** Mammography screening, Informed choice, Crowd-figure-pictograms, Numeric knowledge

## Abstract

**Objective:**

To evaluate the effect of crowd-figure-pictograms on women’s numeric knowledge about mammography screening in a three-armed parallel randomised controlled trial.

**Results:**

552 women were randomised to receive (1) non-numeric information (n = 192), (2) non-numeric and numeric information (n = 186), or (3) non-numeric and numeric information complemented by crowd-figure-pictograms (n = 174). Baseline numeric knowledge was low (control 0.61, numeric 0.66, and pictogram 0.51 on a scale ranging from 0 to 5). Women in the crowd-figure-pictogram group had a larger knowledge increase than women in the numeric group (2.42 vs 2.06, *p* = .03). Both groups had significant increases in knowledge compared to the control (0.20, *p* < .001). Providing numeric information in absolute numbers improves knowledge; even more so when crowd-figure-pictograms are added.

*Trial registration* German Clinical Trials Register DRKS00014736, retrospectively registered 11 May 2018

**Electronic supplementary material:**

The online version of this article (10.1186/s13104-018-3437-z) contains supplementary material, which is available to authorized users.

## Introduction

In mammography screening, informed choice is of special importance because it is unclear whether benefits outweigh harms [1]. Fewer women die of breast cancer when they participate in mammography screening, but screening comes with side effects [1, 2]: anxiety, false alarm, false reassurance, biopsies, overdiagnosis and overtreatment [3]. Many complications are caused by incomplete or incomprehensible information and not by the screening process itself. Symptoms may be ignored because of a false sense of security following a negative result, and health service staff may be blamed unfairly for inherent screening characteristics [4]. More information is likely to reduce those consequences. To achieve an informed choice, knowledge of possible screening outcomes and their likelihood is a prerequisite [5].

Despite guidelines and ethical considerations, risks are often not well communicated [6]. Two problems emerge in current brochures: (1) the completeness of information, and (2) the presentation of information. Often, women receive biased information that aims at encouraging participation and neglects major harms [7]. Health specific sources mostly do not explain the size of benefit and they use relative risk reduction rather than absolute risk reduction [8, 9] even though relative risk is essentially meaningless when presented in isolation. Not surprisingly, more than 9 in 10 women overestimate mortality reduction as a result of mammography screening and consulting health pamphlets tends to increase this overestimation [5].

The brochure ‘Screening for Breast Cancer with Mammography’ [10] was developed to provide understandable evidence-based information for women deciding about whether to attend mammography screening. It includes non-numeric as well as numeric information in absolute numbers. Numeric information has been shown to lead to a more accurate risk perception [11]. People receiving evidence-based information with absolute risks were less likely to be influenced by physician recommendations than people receiving non-evidence-based information that reported only benefits described as relative risks [12]. This indicates that numeric information in absolute numbers is understandable—but there is still room for improvement.

Crowd-figure-pictograms—also called icon-arrays or pictographs—improve understanding of probability compared to verbal information [13] as well as accuracy of risk perception [14]. This effect was found irrespective of level of numeracy [14]. Crowd-figure-pictograms have also been shown to improve medical decision making [15]. In a previous study on lung cancer screening [16], presentation of numbers and crowd-figure-pictograms in combination resulted in higher knowledge levels than numbers alone. However, it remains unclear whether knowledge about mammography screening can be improved by depicting a crowd-figure-pictogram for each numeric information item.

The objectives of this study were to analyse whether numeric information in absolute numbers and numeric information complemented by crowd-figure-pictograms are effective in increasing women’s numeric knowledge about mammography screening compared to a control group and whether there is added benefit in crowd-figure-pictograms compared to only numeric information.

## Main text

### Methods

#### Study design and participants

This study was designed as a 3-armed parallel randomised controlled trial with equal allocation ratio. The arms of the trial were ordered according to information content and presentation: (1) Control intervention (only non-numeric information); (2) Numeric intervention (non-numeric and numeric information); (3) Crowd-figure-pictogram intervention (non-numeric and numeric information complemented by crowd-figure-pictograms).

Following the approval of the protocol by the School of Health and Related Research’s Research Ethics Committee (The University of Sheffield, United Kingdom), an e-mail containing the link to the study was distributed through the University of Sheffield staff- and student-volunteer-e-mail-lists in February 2011. All women were eligible to participate as they either were targeted by the mammography screening programme or would be eligible for the programme in the future (i.e., all female staff and students of the University of Sheffield, UK, were eligible to participate).

#### Procedure

Informed consent was obtained and discontinuing the survey was possible at any time. Participants enrolled themselves and were randomly assigned to one of the three parallel groups according to their month of birth through conditional branching. Which month led to which intervention had been randomly assigned through a computer-generated randomisation sequence.

The following parts were presented consecutively in a single online session: (1) Demographic questions, (2) mammography knowledge questions (preintervention), (3) intervention/active control (a disclaimer before the intervention stated that the data was taken from the brochure ‘Screening for Breast Cancer with Mammography’ [10]), and (4) mammography knowledge questions (postintervention).

#### Mammography questions

The multiple choice mammography questions were tailored to the above-mentioned brochure [10]. The concept questions (1–2) served as indicators of whether participants understood the non-numeric parts of the intervention and were accordingly not expected to differ between the groups. The numeric questions (3–7) were based on 2000 women undergoing screening for 10 years. Eight answer options covered the whole range from 0 to 2000 to avoid hinting the answer through the categories given. Participants were encouraged to give their best guess.

#### Control

The control group received a text about mammography screening, which provided only non-numeric information. It consisted of excerpts from the above-mentioned brochure [10] and provided information about the purpose of screening, improved survival, overdiagnosis, overtreatment, false alarm, pain at examination and false reassurance.

#### Numeric intervention

The numeric intervention group received a text in which the risk of each outcome was expressed as event rate per 2000 women screened regularly for 10 years.

#### Crowd-figure-pictogram intervention

The crowd-figure-pictogram intervention group received the same information as the numeric intervention group but each numeric information item was amended by a crowd-figure-pictogram, which consisted of 2000 female person icons.

#### Outcome measures

The primary outcome measure was increase in numeric knowledge. Answers were scored using an a priori specified marking scheme, following similar approaches [17–19]. Correct responses were assigned 1 point. ‘Don‘t know’ answers and missing values were coded as wrong answer. A composite score of questions 3–7 (possible score range 0–5 points) was assigned to every participant pre- and postintervention, and the difference was calculated. Background variables (age, faculty, role at the university, nationality and previous experience with cancer and breast cancer) were assessed.

#### Statistical analysis

Assuming a difference of means of 0.17, derived from changes in knowledge about purpose of screening and lifetime risk [20], the calculated sample size for each group was $n=428$ (two-sided hypothesis testing; type I error rate of 5%; type II error rate of 20%). Data were analysed with SPSS version 23.0 (IBM, Corp., Armonk, NY). To evaluate successful randomisation, possible baseline differences on background variables between trial arms were statistically tested with an $\alpha$ of .15.

A one-way independent analysis of variance (ANOVA) was performed to compare the mean differences between the three groups. Welch’s F was calculated for comparison of several means in the presence of non-homogenous variances [21, 22], $\omega ^2$ as effect size for the ANOVA [23, 24]. Subsequently, planned orthogonal contrasts were performed because specific predictions were present a priori [22, 25]. The first contrast compared the control against both experimental groups, the second contrast compared the numeric group against the crowd-figure-pictogram group. As effect size for the contrasts, *r* was reported [25, 26].

### Results

#### Participation and baseline characteristics

**Fig. 1:**
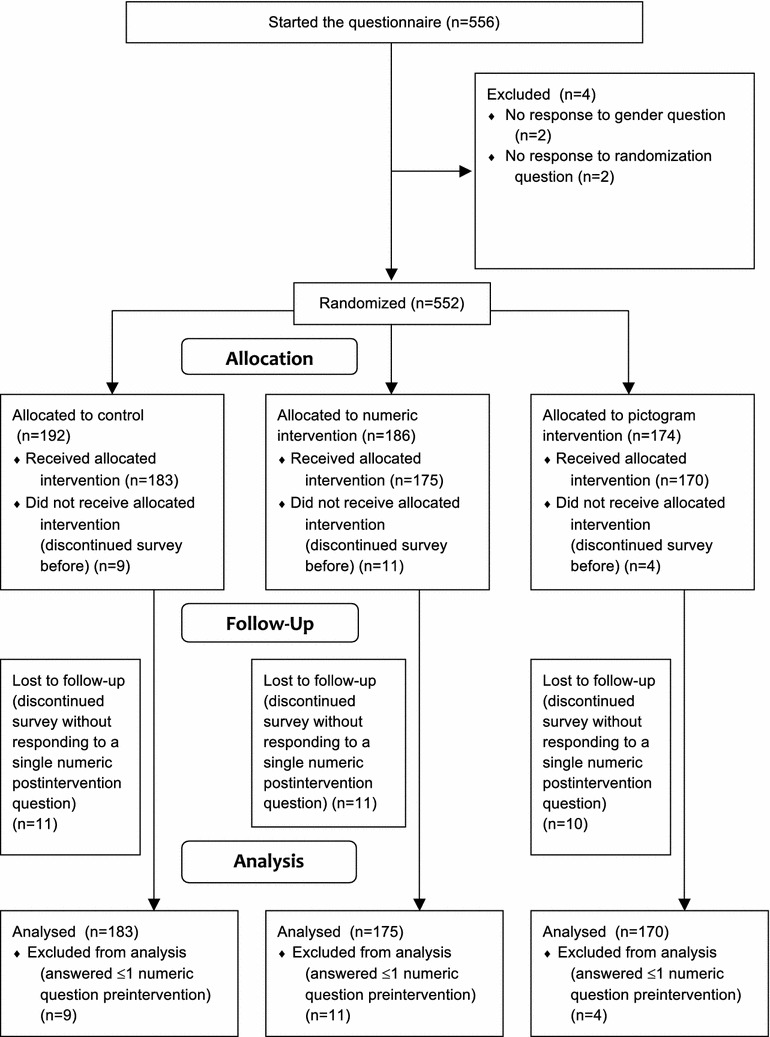
CONSORT flow diagram

556 women started the questionnaire (Fig. 1), 552 answered the randomisation question receiving an allocation to a study group. 24 participants answered neither Question 7 nor more than one of the other numeric questions preintervention, and were assumed to not have received the allocated intervention. Therefore, they were excluded from subsequent analysis. Thus, the analysis was based on $n=528$. Of these, 32 women answered none of the numeric questions postintervention being classified as lost to follow-up, but were nevertheless included in the analysis. Demographic characteristics were similar between groups (see Table 1). $84\%$ were younger than the screening targeted age group. About one in five had had a breast cancer screening within the last 5 years. More than $85\%$ were born in the UK.

**Table 1:** Demographic characteristics of participants, n (%)

	Control	Numeric	Pictogram
Age			
18–49	124 (84.4)	121 (84.0)	119 (84.4)
50–70	23 (15.6)	23 (16.0)	22 (15.6)
Role at university			
Undergraduate	68 (37.4)	64 (36.8)	70 (41.2)
Postgraduate	40 (22.0)	38 (21.8)	31 (18.2)
Staff	62 (34.1)	55 (31.6)	48 (28.2 )
Other	12 (6.6)	17 (9.8)	21 (12.4)
Subject area			
Arts and humanities	23 (14.6)	23 (14.9)	37 (24.5)
Engineering	12 (7.6)	12 (7.8)	9 (6.0)
Medicine, dentistry and health	59 (37.3)	54 (35.1)	45 (29.8)
Science	31 (19.6)	26 (16.9)	29 (19.2)
Social sciences	33 (20.9)	39 (25.3)	31 (20.5)
Country of birth			
UK	153 (86.4)	145 (86.8)	142 (87.7)
Europe	15 (8.5)	13 (7.8)	9 (5.6)
Non-European/overseas	9 (5.1)	9 (5.4)	11 (6.8)
Breast cancer screening within last 5 years			
Yes	39 (21.3)	40 (23.0)	32 (18.8)
No	144 (78.7)	134 (77.0)	138 (81.2)
Breast cancer diagnosis within family			
Yes	66 (36.1)	58 (33.3)	55 (32.4)
No	117 (63.9)	116 (66.7)	115 (67.6)

**Table 2:** Preintervention knowledge

Question	Answer categories	*n* (%)
1. Mammography screening has the following aim	Avoid breast cancer	1 (0.2)
	*Detect breast cancer early*	*520 (98.5)*
	Treat breast cancer	1 (0.2)
	Don’t know	6 (1.1)
2. If the screening result is negative (no abnormality on the X-ray), this means there is definitely no cancer	Correct	69 (13.1)
	*Not correct*	*385 (72.9)*
	Don’t know	73 (13.8)
3. Imagine 2000 women are screened regularly for 10 years. How many will experience pain during the screening?	None	79 (15.0)
	1–4	28 (5.3)
	5–12	23 (4.4)
	13–33	36 (6.8)
	34–90	60 (11.4)
	91–245	66 (12.5)
	246–665	63 (11.9)
	*666–2000*	*67 (12.7)*
	Don’t know	103 (19.5)
4. Imagine 2000 women are screened regularly for 10 years. How many will experience a false alarm?	None	4 (0.8)
	1–4	14 (2.7)
	5–12	50 (9.5)
	13–33	82 (15.5)
	34–90	115 (21.8)
	*91–245*	*125 (23.7)*
	246–665	40 (7.6)
	666–2000	8 (1.5)
	Don’t know	89 (16.9)
5. Imagine 2000 women are screened regularly for 10 years. How many will become breast cancer patients (confirmed by further examinations)?	None	–
	1–4	3 (0.6)
	*5–12*	*21 (4.0)*
	13–33	67 (12.7)
	34–90	89 (16.9)
	91–245	159 (30.1)
	246–665	94 (17.8)
	666–2000	6 (1.1)
	Don’t know	88 (16.7)
6. Imagine 2000 women are screened regularly for 10 years. How many will be treated for breast cancer unnecessarily?	None	136 (25.8)
	1–4	89 (16.9)
	*5–12*	*86 (16.3)*
	13–33	65 (12.3)
	34–90	45 (8.5)
	91–245	7 (1.3)
	246–665	3 (0.6)
	666–2000	1 (0.2)
	Don’t know	93 (17.6)
7. Imagine 2000 women are screened regularly for 10 years. How many will avoid dying from breast cancer?	None	0 (0.0)
	*1–4*	*14 (2.7)*
	5–12	29 (5.5)
	13–33	38 (7.2)
	34–90	86 (16.3)
	91–245	99 (18.8)
	246–665	91 (17.2)
	666–2000	66 (12.5)
	Don’t know	104 (19.7)

Correct answers are italic. *n* = 528

Over 98% knew the aim of mammography screening (Table 2). Approximately a quarter reported the correct number of false alarms. Only $4\%$ knew how many women of the screening cohort were going to become breast cancer patients. A quarter thought there would be no overtreatments. Only $3\%$ knew the correct number of deaths avoided while the largest group thought that 91–245 deaths would be avoided per 2000 women screened regularly for 10 years.

#### Analysis of differences in numeric knowledge

For overall scores on the numeric questions (see the figure in Additional file 1), there was negligible improvement in the control group (difference: $M=0.20$, $SD\pm 0.93$; preintervention: $M=0.61$, $SD\pm 0.73$; postintervention: $M=0.80$, $SD\pm 0.80$) and substantial improvement in the numeric (difference: $M=2.06$, $SD\pm 1.50$; preintervention: $M=0.66$, $SD\pm 0.74$; postintervention: $M =2.73$, $SD\pm$1.34) and pictogram group (difference: $M=2.42$, $SD\pm 1.50$; preintervention: $M=0.51$, $SD\pm 0.76$; postintervention: $M=2.92$, $SD\pm 1.40$). Visual inspection of the histograms and quantile-quantile plots supported normality. For the mean difference on the numeric questions, the variances were significantly heterogeneous in the three groups (Levene’s test; $p<.001$). Therefore, Welch’s F and planned contrasts not assuming equal variances were reported.

There was a significant effect of information type on scores on the numeric questions [$F(2, 323)=187.15$, $p<.001$]. The effect size was large ($\omega ^2=.35$). Planned contrasts revealed that numeric information in any presentational format compared to non-numeric information significantly increased the score on the numeric questions [$t(513)=19.27$, $p<.001$]. Again, the effect size was large ($r=.65$). A crowd-figure-pictogram compared to only numeric information increased the score on the numeric questions significantly [$t(343)=2.19$, $p=.029$] with a small effect ($r=.12$).

### Discussion

Our hypotheses were supported: (1) Non-numeric information and numeric information complemented by crowd-figure-pictograms are effective compared to a control receiving only non-numeric information, and (2) there is added benefit in crowd-figure-pictograms compared to only numeric information. Our finding that numeric information in absolute numbers improves numeric knowledge is in concordance with a literature review which concluded that provision of written information increases knowledge [27]. Contrastingly, in another study, knowledge was not improved for false negatives, recall and interval cancer [20].

The result that crowd-figure-pictograms improve numeric knowledge differs from some published studies. In a review, only one study was found in the category ‘Numerical and graphical vs numerical information only’ and it reached a low method score [28]. Conversely, the present findings are supported by a study on decision aids for 70-year-old women [17]. Similar results were obtained for 40-year-old women [29]. This similarity of outcomes might have to be interpreted with caution, as the two described studies also included a value clarification exercise possibly interacting with the effects of crowd-figure-pictograms on knowledge. Regarding the type of icons used in the crowd-figure-pictograms in previous research, person icons were not only most preferred but anthropomorphic icons have been shown to lead to improved risk recall [30]. This is in line with our finding of improved numeric knowledge following a crowd figure pictogram using person icons.

Additional crowd-figure-pictograms yielded a beneficial effect on knowledge and constitute an effective format of risk communication. Essentially, the present study adds the evaluation of the added benefit of including crowd-figure-pictograms in information materials designed for women in the age group targeted by population based screening programs. Our results suggest that crowd-figure-pictograms in combination with numeric information in absolute numbers lead to a larger knowledge increase than achievable through solitary presentation of numeric information in absolute numbers.

## Limitations

The baseline knowledge levels were probably not representative for knowledge levels in the population of UK University staff and students even though our sample was large. This only affected the generalisability but not the internal validity of this randomised controlled trial.

In the evaluable participant analysis, missing values were coded as wrong answers. This allowed inclusion of cases lost to follow-up and participants not responding to all preintervention questions, constituting a conservative approach. Since all cases lost to follow-up after the intervention were included, the danger for overestimation of an effect can be assumed as reasonably low.

Even though the decision was not relevant for most women, since they were not in the screening-targeted age group, a broad age distribution was covered. Informing women about mammography screening is not a task that only becomes relevant with the onset of screening age, since, prior to that age, opportunistic screening is possible and an attitude towards screening may be formed.

## Additional files

**Additional file 1.** Scores on the numeric questions by intervention group. Error bars indicate 95% confidence intervals.

## Abbreviations

ANOVA: analysis of variance
UK: United Kingdom
