# Supplementary figures and images for: Crowd-figure-pictograms improve women’s knowledge about mammography screening: results from a randomised controlled trial
Source: BMC Res Notes. 2018 May 21;11:332. doi: 10.1186/s13104-018-3437-z (PMC5963070; doi:10.1186/s13104-018-3437-z)

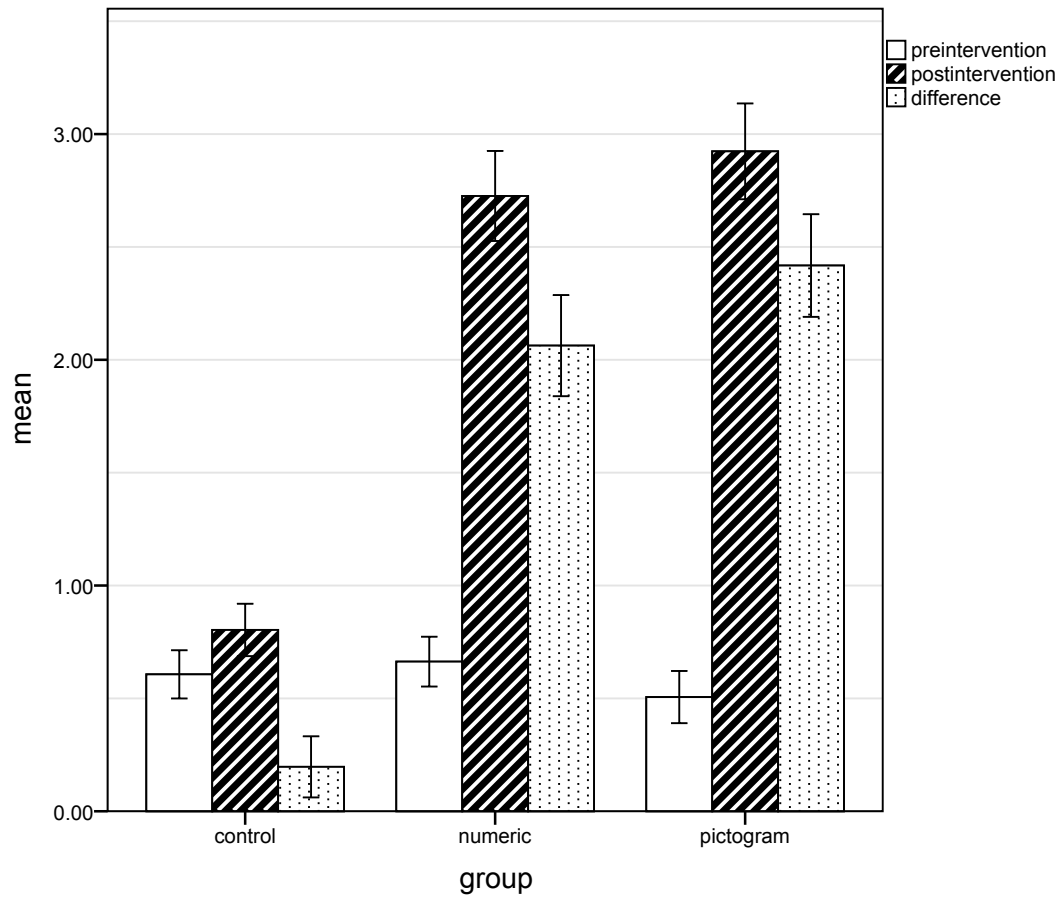

Supplement: Supplementary file 1 — Additional file 1. Scores on the numeric questions by intervention group. Error bars indicate 95% confidence intervals. [file 13104_2018_3437_MOESM1_ESM.pdf]
